# Abdominopelvic Actinomycosis—The Diagnostic and Therapeutic Challenge of the Most Misdiagnosed Disease

**DOI:** 10.3390/life12030447

**Published:** 2022-03-17

**Authors:** Alin Mihai Vasilescu, Eugen Târcoveanu, Cristian Lupascu, Mihaela Blaj, Corina Lupascu Ursulescu, Costel Bradea

**Affiliations:** 1 Department of Surgery, St. Spiridon Emergency University Hospital, Grigore T. Popa University of Medicine and Pharmacy, 16 University Street, 700115 Iassy, Romania; etarcov@yahoo.com (E.T.); cristian.lupascu@umfiasi.ro (C.L.); costel.bradea@umfiasi.ro (C.B.); 2 Department of Intensive Care, St. Spiridon Emergency University Hospital, Grigore T. Popa University of Medicine and Pharmacy, 16 University Street, 700115 Iassy, Romania; mihaela.blaj@umfiasi.ro; 3 Department of Radiology, St. Spiridon Emergency University Hospital, Grigore T. Popa University of Medicine and Pharmacy, 16 University Street, 700115 Iassy, Romania; corina.ursulescu@umfiasi.ro

**Keywords:** abdominopelvic actinomycosis, colon cancer, pelvic tumor, inflammatory tumor, hepatic actinomycosis

## Abstract

Abdominopelvic actinomycosis is a rare chronic or subacute bacterial infection caused by *Actinomyces israelii*, a Gram-positive anaerobic bacterium that normally colonizes the digestive and genital tracts, clinically presented as an inflammatory mass or abscess formation. Methods: We reviewed the medical records of the patients from our clinic with abdominopelvic actinomycosis who underwent surgery between 2002 and 2022. In this period, 28 cases (9 men and 19 women) were treated. The mean age was 43.36 years and they were hospitalized for abdominopelvic tumors or inflammatory tumors in 15 cases and inflammatory disease in 13 cases. Results: Causes of actinomycosis in the studied group were an intra-uterine contraceptive device in 17 cases, foreign bodies in 2 cases, diabetes in 4 cases, stenting of the bile duct in 1 case, and immunodepression. For 6 patients, we performed surgery by open approach and for 21 patients by a laparoscopic approach. For nine patients, abdominopelvic actinomycosis had been mimicking a colon malignancy (cecum and ascending colon, four cases; transverse colon, two cases; and on the greater omentum, three cases) and for six patients, a pelvic tumor (advanced ovarian cancer). After surgery the patients underwent specific treatment with antibiotics, with good results. In two cases we discovered and treated hepatic actinomycosis, one case by a laparoscopic approach and one case by a percutaneous approach. In our lot we noticed three recurrences that required reintervention in patients who had had short-term antibiotics due to non-compliance with treatment out of four such cases. Conclusions: For abdominopelvic malignancies, actinomycosis should be included in the differential diagnosis, as well as for inflammatory bowel diseases and bowel obstructions. We have a wide range of patients considering the rarity of this condition. Long-term antibiotics are necessary to prevent recurrence.

## 1. Introduction

Abdominopelvic actinomycosis is a rare chronic or subacute bacterial infection caused by *Actinomyces israelii*, a Gram-positive anaerobic bacterium that normally colonize the digestive and genital tracts; it is often confused with a tumor or manifests as an inflammatory tumor, and can also form abscesses. The evolution is indolent and latent with local inflammatory extension without limitation to a single organ [1,2]. The *Actinomyces* comprises a diverse phylogenetic group of facultative anaerobic or microaerophilic bacilli which are non-sporulated, immobile, and which in vivo may have an apparently branched appearance (resulting in confusion with fungi). The incubation is slow, requiring at least 10 days before conclusion of a negative culture by reason of growth of *Actinomyces* (at least 5 days up to 15–20 days) [3,4,5,6].

## 2. Methods

We conducted a retrospective study on the patients with abdominopelvic actinomycosis who underwent surgery between 2002 and 2022. In this period, 28 cases (9 men and 19 women) were treated in our clinic. We analyzed the medical records of the patients confirmed to have abdominopelvic actinomycosis, including clinical, pathological, microbiological, and therapeutic data. The mean age was 43.36 ± 19.14 years (range between 18 and 64 years). In this study, we describe different clinical features, elements for the diagnosis, and current treatment.

## 3. Results

Patients were hospitalized with abdominopelvic tumors (15 cases) or inflammatory disease (13 cases). We identified an increase of the pelvic actinomycosis associated with long-term (mean of 6.48 years, range from 5 to 11 years) intra-uterine contraceptive devices (IUDs). The other factors that were identified in our group were diabetes mellitus in four cases, foreign bodies in two cases represented by a toothpick in one case and gallstones lost in the peritoneum in another case, stenting of the bile duct in one case, and immunodepression or immunosuppression. Most cases were admitted because they mimicked a complicated abdominopelvic malignant tumor from colon cancer or advanced ovarian cancer. All these cases were admitted in emergency. A clinical exam revealed in most cases a distended abdomen with diffuse tenderness, a tumor with imprecisely delimited borders, sensitive to palpation and deep organ adhesion (42.3%), abdominal pain (76.9%), fever (61.5%), and weight loss (50%). The mean duration of chronic or subacute symptoms was 2.8 ± 2.4 months (range from 1 to 14 months) and for cases operated in emergency was 11.7 ± 9.2 days (range from 7 to 21 days).

**Figure 1:**
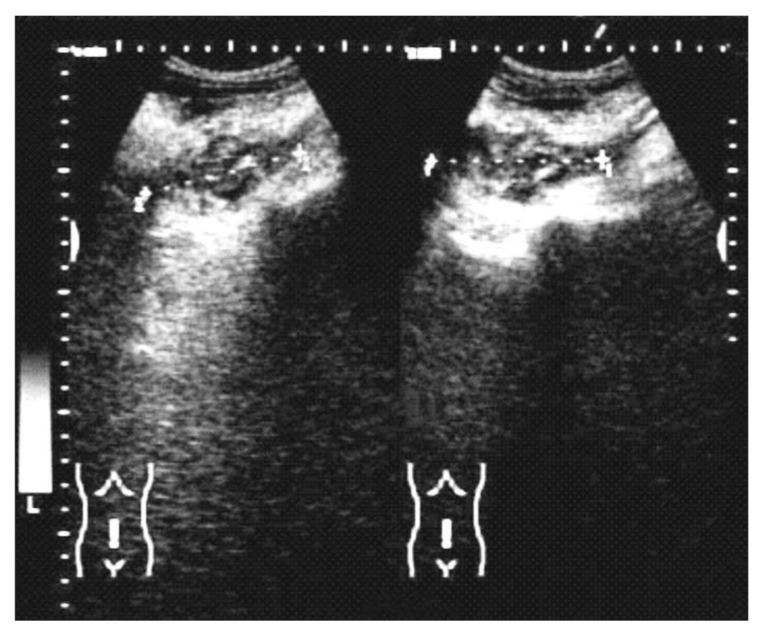
Abdominal ultrasound discovered a heterogeneous abdominal tumor and peritumoral fluid.

**Figure 2:**
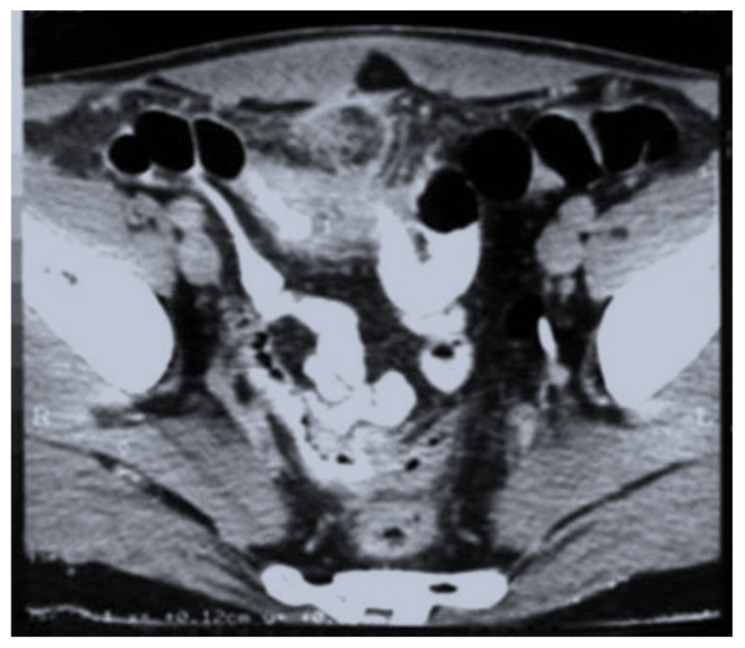
CT scan—heterogeneous mass involving distal ileum; inflammatory changes of the mesentery were also observed.

The disease was rarely suspected on imaging and was usually noncontributory to the positive diagnosis. The abdominal ultrasound (Figure 1) described collections and abscesses found in the abdomen and pelvis, and there was also inflammatory involvement or a solid, heterogeneous mass, surrounded by collections confirmed by CT findings of the abdomen and pelvis (Figure 2), which showed the infiltrative nature of the disease, a heterogeneous mass involving a distal ileum, cecum, and ascending or transverse colon, and an inflammatory mass involving the ovaries for a pelvic tumor. Lab work revealed an elevated white count and anemia, and tumor markers were normal or with slightly modified values in most cases. We did not use polymerase chain reaction (PCR) for diagnosis.

For 6 patients, we performed surgery by and open approach and for 21 patients by a laparoscopic approach.

**Figure 3:**
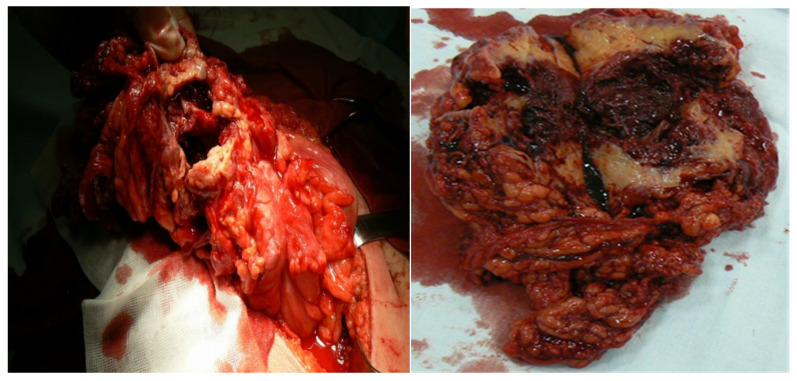
Transverse colon actinomycosis, intraoperative view and operative specimen on the section.

**Figure 4:**
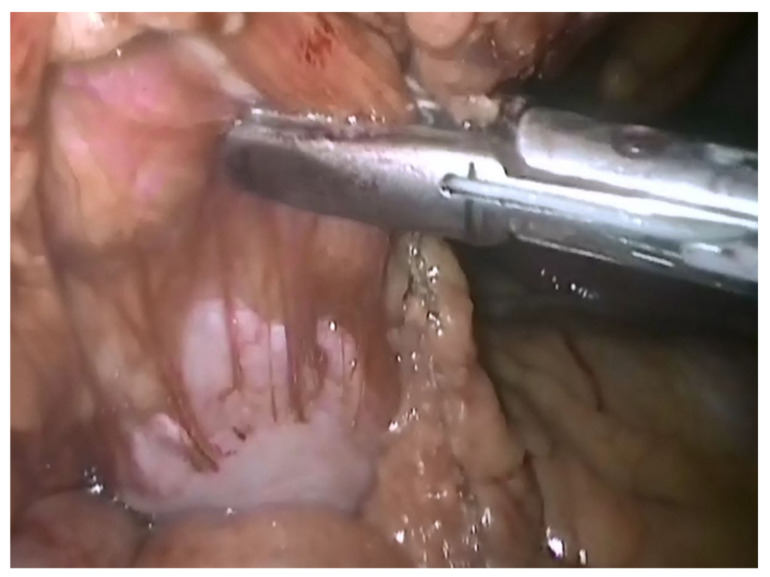
Intraoperatory view—laparoscopic resection of a large omentum mass, with adhesions to the parietal peritoneal serosa.

In nine cases, with the suspicion of colic tumors, the lesions were located on the ascending colon for four patients, transverse colon for two patients, and on the greater omentum for three patients. Imaging was highly suggestive for occlusive colon tumors. We performed a right hemicolectomy (Figure 3) with ileo-transverse anastomosis (four cases, one by laparoscopic approach), a segmental colectomy (two cases) with colo-colic anastomosis, and a laparoscopic resection of an omental tumor in three cases (Figure 4). We discovered that the adjacent mesentery had inflammatory changes in all cases. In two cases the tumors had adherence to deep muscles, the peritoneum, and the abdominal wall, and ablation of the invasion zone was necessary.

Six patients mimicking an advanced ovarian cancer with a history of right iliac fossa pain, mild fever, and clinical examination revealed several pelvic masses and the presence of bilateral ovarian masses along with peritoneal carcinomatosis observation and retroperitoneal lymphadenopathy was confirmed by a CT scan or magnetic resonance scan. Bilateral salpingo-oophorectomy and an omentectomy were performed in six cases after careful dissection of the tumoral mass off the bowel. The hysterectomy and lymphadenectomy were not attempted due to consideration of the severe inflammation and advanced tumor stage.

In two cases we treated hepatic actinomycosis discovered by abdominal CT, one case by a laparoscopic approach (we did not identify any cause or predisposing factors) and one case by a percutaneous approach (the predisposing factor was stenting of the bile duct). A surgical biopsy and percutaneous biopsy sample showed several sulfur granules in the background of an organizing abscess.

**Figure 5:**
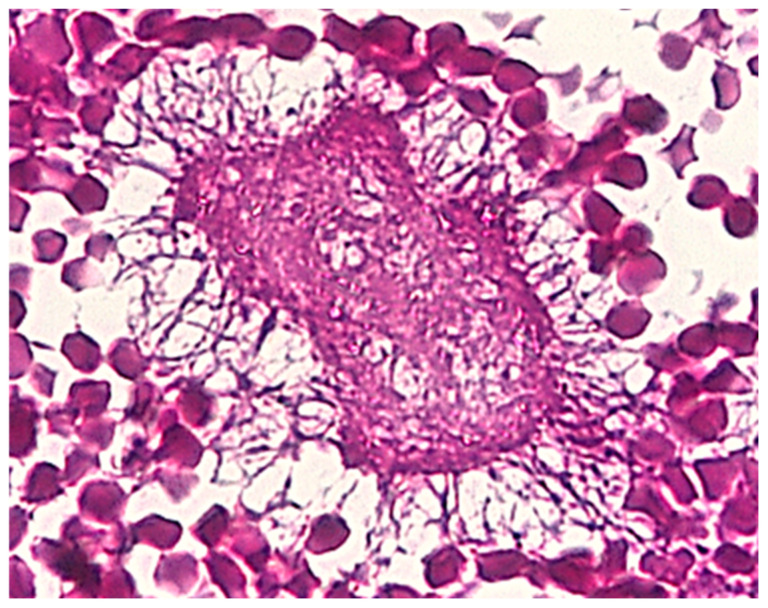
Abdominal actinomycosis. PAS Positive—Actinomyces israelii Colony, X 20.

The disease was determined after identifying colonies of *Actinomyces israelii* on pathology (Figure 5) or bacterial culture from surgically drained pus (15 cases). The histopathology discovered a granulomatous inflammation located in the intestinal wall, in the omentum, and of the parietal peritoneum with confluent serosa and mesentery abscesses. Frozen sections were negative for malignancy and colonies of *Actinomyces israelii* were identified. The histopathology for pelvic tumors was characterized by the presence of multiple confluent abscesses, with numerous colonies of actinomycoses with sulfur granules, inflammatory infiltrates rich with foamy macrophages and fibrin.

All patients were treated with antibiotics (intravenous penicillin for 4–6 weeks and after with amoxicillin oral) with a favorable postoperative course. The mean duration of treatment was 6.5 months ± 4.6 months ranging from 3 weeks to 12 months, with regard to the clinical and laboratory symptoms. In our lot we noticed three recurrences that required reintervention in patients who had had short-term antibiotics due to non-compliance with treatment out of four such cases.

There were no poor results for patients receiving long-term antibiotics; all patients were free of recurrence. The main characteristic of the patients with abdominopelvic actinomycosis from our study are described and synthesized in Table 1.

## 4. Discussion

The actinomycosis was discovered by Israel in 1879. Actinomycosis occurs worldwide, with higher prevalence rates in areas with low socioeconomic status and poor dental hygiene. Except for pelvic actinomycosis, middle-aged male patients are affected more commonly than female patients. We have a wide range of patients considering the rarity of the condition, which is still becoming more common. We noticed that in our group, if we exclude cases with actinomycosis after IUDs, the ratio is in favor of male patients.

A normal inhabitant of the mouth, *Actinomyces israelii* is a commensal inhabitant of the oral cavity and intestinal tract [4] but acquires pathogenicity through the invasion of breached or necrotic tissue. It is an opportunistic infection, usually in association with bacterial invasion. It tends to follow a break in normal mucosal barriers. As the infection progresses, granulomatous tissue, extensive reactive fibrosis and necrosis, abscesses, draining sinuses, and fistulas are formed [5]. Infection involving the cervicofacial area is most common, followed by abdominal and pelvic involvement and thoracic involvement [6]. The loss of the integrity of the mucous membrane of the oral cavity, caused by extractions, endodontic treatment, bone and dental fractures, and periodontal disease can cause infection with these microorganisms [7].

This infection usually spreads locally slowly, and it may take months before any symptoms appear [8]. It is not able to cross normal mucosal barriers and, therefore, opportunistic infections can occur only in the context of underlying local disease. The destruction of the mucosal barrier by trauma, operations, immunosuppression, a foreign body, or a history of perforated viscera are recognized as predisposing factors.

Sporadic and genitourinary impairment may occur with renal or perirenal tumors [9]. Thoracic disease can result from either direct extension into the mediastinum from a cervico-facial infection or transdiaphragmatic or retroperitoneal dissemination from the abdomen. Hematogenous spread to the lung is also described. Intra-abdominal actinomycosis often has an abdominopelvic form that usually occurs after impaired intestinal integrity (usually appendicectomy, perforated bowel, diverticulitis, gastrointestinal surgery, endoscopic procedures, or trauma), but sometimes no cause is identified [10].

For the large bowel, the most common site of actinomycosis is the cecum and ascending colon. It can be confused with colon cancer as in transverse colon localization it is less affected [11,12,13], as was also described in our cases. There are also cases with actinomycosis with localization of the sigmoid colon. At this level it can mimic a diverticulitis or colon cancer [14]. Also, tuberculosis, Crohn’s disease or other inflammatory diseases may be confused with actinomycosis. Imaging investigations (US, CT, MRI) confirm the presence of a mass or abscess but they are not able to distinguish between actinomycosis and other pathologies such as malignancy, Crohn’s disease, diverticulitis, appendicitis, pelvic peritonitis, or tuberculosis, because in this disease solid masses with focal low-attenuation areas were more frequently found than cystic masses with thickened walls [15].

Predisposing risk factors reported for actinomycosis are recent surgery, abdominal trauma, endoscopic procedures, visceral perforations (large or small bowel, appendix, diverticulum) [16], foreign bodies (as in our cases: fish bones, toothpicks) [17], gallstones lost in the peritoneum [18], immunosuppressed patients, and diabetes mellitus. Predisposing risk factors for pelvic genital actinomycosis were reported in association with IUDs, and the colonization rate increases with the duration of its maintenance (minimum 5 years, although there are studies that describe cases even after 1 year [19]). Pelvic actinomycosis is considered to be a rare disease, although the use of IUDs can promote its appearance. It is recommended that IUDs be changed periodically, every 3–5 years, to limit the occurrence of this disease [20].

Clinically abdominopelvic actinomycosis has various nonspecific symptoms. The most common symptoms are palpable mass, fever, weight loss, abdominal pain or discomfort, nausea, and vomiting as in our cases. The severity of the disease is accordingly determined by the quantity of proteolytic enzymes secreted, associated anaerobic bacteria, and, importantly, the immune status of the host, which explains the wide variation in clinical severity, which may range from chronic asymptomatic to fulminant forms [15].

Preoperative diagnosis of abdominal actinomycosis is difficult. An accurate diagnosis is always obtained by histological or microbiological examination. There is the possibility of a delayed diagnosis because of an insufficient incubation period and the lack of the appropriate anaerobic media. Recognition is important because successful treatment requires combined surgery and prolonged penicillin treatment [20,21]. As the data in the literature show, and in our series, where there were only two cases that received a preoperative diagnosis, the diagnosis of actinomycosis is a surprise upon histopathological examination and the treatment in emergency often makes it difficult to conduct additional preoperative examinations.

The clinical findings of actinomycosis may be present in other chronic diseases, such as granulomatous diseases (Crohn’s disease) [22] or malignant lesions [23]. Unfortunately, and in our series of cases, this condition is diagnosed in most cases postoperatively at the pathological examination. The better preoperative diagnosis would allow antibiotic treatment, and surgical treatment should be reserved only for complications. It is very difficult to diagnose actinomycosis preoperatively, because imaging studies are also nonspecific. CT scan results of an infiltrating abdominopelvic mass without border limits and increased heterogeneous contrast may suggest actinomycosis, especially in patients with fever, leukocytosis, or predisposing factors. Because of the size of the bacterium, it usually does not spread via the lymphatic system, so that regional lymphadenopathy is rare [24].

In the case of an abdominal abscess, the preoperative diagnosis of actinomycosis can be confirmed by percutaneous ultrasound-guided fine-needle aspiration [25]. Frequently, final diagnosis is only made postoperatively and is based on culture of the organisms, or the presence of “sulfur granules” in the specimen.

For severe infection, an initial course of intravenous penicillin G (10 to 20 million units/24 h) is indicated [26]. Administration of penicillin G as a continuous infusion may make administration easier; Ceftriaxone (1 to 2 g every 24 h) is a reasonable alternative that can also be more easily administered in an outpatient setting [27].

The indications for surgery are when the disease is persistent or complications occur. Medical treatment with an antibiotic is tried first in uncomplicated cases [28]. Surgery is often indicated due to difficulty in diagnosis and is also justified in some severe cases. Medical treatment with antibiotics and surgery are also indicated in the case of a complicated actinomycosis. This combination of surgery and antibiotics results in healing in the majority of cases [14], as in our group, and relapses respond to medical treatment by healing without surgery. Surgical ablation of the mass with drainage, supplemented with the long-term administration of antibiotics, is recommended especially in patients in which the lesion mimics abdominopelvic cancer. *Actinomyces* are usually extremely susceptible to beta-lactams. As for the antibiotic treatment of actinomycosis, penicillin G followed by oral penicillin or amoxicillin for up to 12 months are considered the drugs of choice [29], and other antibiotics such as erythromycin, clindamycin, or tetracycline can be given, especially in cases with penicillin allergy [30]. Drug resistance is not a problem in actinomycosis. Broad-spectrum antibiotics, such as piperacillin–tazobactam, imipenem, and meropenem, can be used, but these should be limited to avoid the acquisition of resistant flora. Fluoroquinolones (ciprofloxacin and moxifloxacin) are usually considered to be ineffective and it is not useful to combine amoxicillin with beta-lactam inhibitors, except if there is an infection with Enterobacteriaceae [31,32].

Findings which are highly suggestive for the diagnosis can be a chronic granulomatous infection with sulfur granules. Pathology exams reveal yellowish sulfur granules (a conglomeration of bacteria trapped in biofilm) in most cases, necrosis, and filamentous Gram-positive fungal-like pathogens [6,31].

In Table 2, we present several publications with cases and the problems of diagnosis and treatment that involve abdominopelvic actinomycosis, problems that have been encountered in our cases as well [8,14,33,34,35,36,37,38,39,40,41,42,43].

## 5. Conclusions

Abdominopelvic actinomycosis is rare, with a satisfactory prognosis in the case of early diagnosis, and should be included in the differential diagnosis of abdominal and pelvic tumors. However, it should be considered in the differential diagnosis of inflammatory bowel diseases and bowel obstructions without a clear cause as well. Preoperative diagnosis is difficult. Clinical and imaging findings are likely to suggest malignancy and bacterial cultures and pathology are necessary for exact diagnosis. Precipitating factors of abdominal actinomycosis may be gastrointestinal surgery for acute appendicitis, diverticulitis, and intra-uterine contraceptive devices in pelvic tumors of women, which in some cases is presented as advanced ovarian cancer. Surgery is still needed to improve medical treatment or to treat pelvic abscesses.

Extensive resection is useful therapeutically for the purpose of debulking and treatment with antibiotic for 6 months is necessary to cure the disease and prevent recurrence. Postoperative follow-up is necessary to identify recurrences, especially in cases with short-term antibiotic administration.

## Figures and Tables

**Table 1:** The main characteristics of the 28 patients with abdominopelvic actinomycosis.

Age	43.36 ± 19.14 years (18–64 years)
Gender	9 male/19 female patients
Predisposing factors	N (cases) (%)
Long-term IUDs	13/28 (46.4%)
Diabetes	5/28 (17.8%)
Foreign bodies	
Toothpick	1/28 (3.5%)
Gallstones lost in the peritoneum	1/28 (3.5%)
Stenting of the bile duct	1/28 (3.5%)
Immunosuppression	3/28 (10.7%)
Diverticulitis	3/28 (10.7%)
Oral diseases associated	5/28 (17.8%)
Gastroesophageal reflux associated	9/28 (32.1%)
No predisposing factors	1/28 (3.5%)
Clinical features	N (cases) (%)
Distended abdomen with tenderness	11/28 (39.2%)
Tumor palpable	4/28 (14.2%)
Deep organ adhesion	12/28 (42,3%)
Abdominal pain	18/28 (76,9%)
Fever	17/28 (61,5%)
Weight loss	14/28 (50%)
Anemia	13/28 (46.4%)
Leukocytosis	23/28 (82.14%)
The mean duration of symptoms	
Chronic or subacute symptoms	2.8 ± 2.4 month (1–14 months)
Emergency	11.7 ± 9.2 days (7–21 days)
Imaging (US, CT) characteristic	N (cases) (%)
Intraperitoneal collections	11/28 (39.2%)
A heterogeneous mass involving the colon	6/28 (21.4%)
Omental mass	3/28 (10.7%)
Inflammatory mass involving the ovaries	6/28 (21.4%)
Right liver abscesses	2/28 (7.1%)
Treatment	N (cases) (%)
Open approach	6/28 (21.4%)
Right hemicolectomy	3/6 (50%)
Segmental colectomy	2/6 (33.3%)
Drainage of peritoneal abscess	1/6 (17.6%)
Laparoscopic approach	21/28 (21.4%)
Omental laparoscopic resection	3/21 (14.2%)
Right hemicolectomy	1/21 (4.7%)
Bilateral salpingo-oophorectomy	6/21 (28.5%)
Drainage of peritoneal abscess	11/21 (5.2%)
Drainage and biopsy of the liver abscess	1/21 (4.7%)
Radiologic percutaneous approach of the liver abscess	1/28 (3.5%)
Postoperative treatment—intravenous penicillin for 4–6 weeks (12 to 20 million units daily in divided doses every four to six hours) + amoxicillin oral	6.5 months ± 4.6 months (3 weeks–12 months)
Recurrences	3/28 (10.7%)

**Table 2:** Publications of cases with abdominopelvic actinomycosis.

Author/Year	Total Cases	Involved Sites	Mean Age	Gender, M/F	Predisposing Factors	Leukocytosis	Presumptive Diagnosis	Final Diagnosis	Treatment
Yegüez JF et al., 2000 [33]	1	Rectosigmoid and cecum	49	F	−	−	Tumor of colon	Histologic diagnosis of surgical specimen	Resection and colostomy + actinomycosis medication
Cirafici L et al., 2002 [34]	1	Left colon	56	F	IUD	+	Tumor occlusive of colon	Histologic diagnosis of surgical specimen	Colostomy + actinomycosis medication
Chelli D et al., 2008 [35]	5	Pelvic inflammatory disease	39.2	F	IUD	+	Pelvic inflammatory disease	Culture of the microorganisms	Drainage + actinomycosis medication
Privitera A et al., 2009 [14]	1	Left colon	67	M		+	Abscessed tumors of the sigmoid colon	Histologic diagnosis of surgical specimen	Hartmann’s procedure + actinomycosis medication
Lim KT et al., 2010 [36]	1	Abdominal wall mass that extended from the dome of the bladder	26	M	−	+	Urachal tumor	Histologic Diagnosis of Surgical Specimen	Partial cystectomy with abdominal wall mass excision + actinomycosis medication
Marret H et al., 2010 [37]	11	Ovary, colon, pelvic inflammatory disease		F	IUD	+	Pelvic inflammatory disease, ovarian cancer, bowel obstruction, acute peritonitis	Culture of the microorganisms, histologic diagnosis of surgical specimen	Total abdominal hysterectomy; salphingo-oophorectomy + actinomycosis medication
Sung HY et al., 2011 [8]	23	Appendix (n = 5), ovarian mass (n = 5), abdominal wall mass (n = 4), colonic mass (n = 4), small bowel mass (n = 2), uterus mass (n = 2), and liver	47.8	5 M/18 F	IUD, total abdominal hysterectomy, Caesarean section, a fish bone-induced rectal perforation polypectomy-induced microperforation, peritoneal dialysis, cholecystectomy with T-tube drainage	+	Acute appendicitis (n = 10), pelvic inflammatory disease (n = 8), and acute tubo-ovarian abscess (n = 4), ovarian cancer, or colon cancer	Histologic diagnosis of surgical specimen	Right hemicolectomy; left hemicolectomy; total abdominal hysterectomy; salphingo-oophorectomy + actinomycosis medication
Jabi R et al., 2020 [38]	1	Left colon	48	M	−	+	Tumor of the left colon	Histologic diagnosis of surgical specimen	Left segmental colectomy with latero-lateral mechanical anastomosis + actinomycosis medication
Asiri BI et al., 2020 [39]	1	Appendix	38	M	−	+	Acute appendicitis and cecum tumor	Histologic diagnosis of surgical specimen	Appendectomy and laparoscope-assisted ileocaecal resection with ileocolic anastomosis + actinomycosis medication
Kim S et al., 2020 [40]	1	Peritoneal and pelvic masses	47	F	IUD	−	Peritoneal carcinomatosis	Histologic diagnosis of surgical specimen	Peritoneal biopsy + actinomycosis medication
Pamathy G et al., 2021 [41]	1	Transverse and descending colon	40	F	IUD	+	Transverse and descending colon malignancy	Histologic diagnosis of surgical specimen	Left hemicolectomy
Clarrett D et al., 2021 [42]	1	Transverse colon, abdominal wall	66	M	−	−	Transverse colon malignancy	Histologic diagnosis of surgical specimen	Surgical resection of the right upper abdominal wall mass en bloc with right hemicolectomy
Tarzi M et al., 2021 [43]	1	Abdominal wall	59	M	Type 2 diabetes	−	Abdominal wall tumor	Histologic diagnosis of surgical specimen	Surgical excisional biopsy

## Data Availability

The data published in this research are available on request from the first and last author and corresponding author.
